# Short‐term tear film stability, optical quality and visual performance in two dual‐focus contact lenses for myopia control with different optical designs

**DOI:** 10.1111/opo.13024

**Published:** 2022-07-08

**Authors:** José Vicente García‐Marqués, Rute Juliana Macedo‐De‐Araújo, Colm McAlinden, Miguel Faria‐Ribeiro, Alejandro Cerviño, José Manuel González‐Méijome

**Affiliations:** ^1^ Optometry Research Group, Department of Optics and Optometry and Vision Sciences University of Valencia Valencia Spain; ^2^ Clinical and Experimental Optometry Research Laboratory (CEORLab) Center of Physics (Optometry), School of Sciences University of Minho Braga Portugal; ^3^ Department of Ophthalmology Royal Gwent Hospital Newport UK; ^4^ Wenzhou Medical University Wenzhou China; ^5^ Eye & ENT Hospital of Fudan University Shanghai China

**Keywords:** light disturbance, multifocal contact lens, myopia control, tear film stability, visual performance, visual quality

## Abstract

**Purpose:**

To assess and compare short‐term visual and optical quality and tear film stability between two dual‐focus (DF) prototype myopia control contact lenses (CLs) having different inner zone diameters.

**Methods:**

Twenty‐eight myopic subjects were included in this randomised, double‐masked crossover study. Refraction, best‐corrected visual acuity (VA) and tear film stability were measured at baseline (i.e., when uncorrected). Subjects were then binocularly fitted with the DF CLs, with only the sensorial dominant eye being assessed. Lenses were of the same material and had inner zone diameters of either 2.1 mm (S design) or 4.0 mm (M design). Visual and physical short‐term lens comfort, over‐refraction, best‐corrected VA, stereopsis at 40 cm, best‐corrected photopic and mesopic contrast sensitivity (CS), size and shape of light disturbance (LD), wavefront aberrations, subjective quality of vision (QoV Questionnaire) and tear film stability were measured for each lens.

**Results:**

Both CL designs decreased tear film stability compared with baseline (*p* < 0.05). VA and photopic CS were within normal values for the subjects' age with each CL. When comparing lenses, the M design promoted better photopic CS for the 18 cycles per degree spatial frequency (*p* < 0.001) and better LD (*p* < 0.02). However, higher‐order aberrations were improved with the S design (*p* = 0.02). No significant difference between the two CLs was found for QoV scores and tear film stability.

**Conclusions:**

Both DF CLs provided acceptable visual performance under photopic conditions. The 4.0 mm inner zone gave better contrast sensitivity at high frequencies and lower light disturbance, while the 2.1 mm central diameter induced fewer higher‐order aberrations for a 5 mm pupil diameter. Both CLs produced the same subjective visual short‐term lens comfort.

**Key points**

- The 4.0 mm central diameter contact lens provided better contrast sensitivity at higher spatial frequencies and lower light disturbance than the 2.1 mm central diameter lens.
- The 2.1 mm central diameter contact lens‐induced lower levels of higher‐order aberrations than the 4.1 mm central diameter lens.
- Neither the short‐term subjective visual comfort nor the tear film stability was influenced by the central diameter of the lens.

## INTRODUCTION

The prevalence of myopia has increased dramatically over the past 60 years.^1^, ^2^, ^3^ A number of methods to control myopia progression in the paediatric population have been developed, including central distance and dual‐focus (DF) contact lenses (CL).^4^ These lenses induce peripheral defocus to reduce off‐axis hyperopia, thereby attenuating a potential stimulus for axial elongation.^1^, ^2^, ^3^, ^4^, ^5^, ^6^, ^7^, ^8^, ^9^, ^10^ It has been reported that DF CLs have an efficacy between 30% and 72% for controlling the spherical equivalent refractive error, and up to 80% for controlling axial length.^8^, ^11^, ^12^, ^13^, ^14^, ^15^

Despite their positive effect on controlling eye growth, DF CLs have the drawback of limiting retinal image quality since the light that enters the pupil is redistributed into different foci.^3^, ^16^, ^17^, ^18^ Generally, DF CLs cause a reduction in contrast sensitivity and induce greater light disturbance and higher‐order aberrations.^1^, ^3^, ^7^, ^16^, ^18^, ^19^ These effects may be pupil‐dependent since the percentage of light distributed at the far and near focus depends upon the pupil diameter. Therefore, the performance of a DF CL is influenced by the zones designed for far and near vision and the pupil diameter exposed to these zones.^19^, ^20^, ^21^, ^22^, ^23^, ^24^, ^25^ It has also been reported that visual quality and patient satisfaction might be improved by adjusting the distance and near zones of the lens.^1^, ^8^, ^20^, ^26^ For instance, Martins et al.^1^ and Talens‐Estarelles et al.^20^ found that the quality of vision was less affected by lenses with larger distance vision areas. However, different types of CLs were used in these investigations.

Another major contribution to the image quality perceived through a CL, irrespective of whether it is DF, is tear film quality. The tear film plays an important role in the fitting and comfort of CLs, and the visual quality of the eye. Therefore, assessing the tear film in CL wearers is vital.^27^, ^28^ When a CL is fitted, it splits the tear film into pre‐lens and post‐lens layers, which may make the tear film less stable.^28^, ^29^, ^30^ A recent study^28^ reported that the concentric ring pattern of a DF CL for myopia control induced a reduction in the tear film stability and comfort when compared to a monofocal lens composed of the same material. This may be caused by the reduced spreading of the pre‐lens tear film across the CL surface due to the abrupt changes in the curvature of the anterior lens surface. The present study extends this work by assessing whether the different inner zones within the concentric ring pattern of a DF CL also affect tear film stability.

To our knowledge, only a few studies to date have assessed the influence of myopia control CLs on visual quality and tear film stability.^1^, ^8^, ^16^, ^28^ Since visual quality, comfort and the tear film play a role in CL fitting and treatment adherence, expanding knowledge on how different designs and materials affect these clinical parameters is highly relevant.^31^

In this study, we compared short‐term visual quality, optical quality, light disturbance, short‐term lens comfort and tear film stability between two prototype myopia control DF CLs, manufactured from the same material.^8^ We hypothesised that these parameters may be influenced by the different CL designs. The present study may help clinicians adjust lens designs based on the patient's needs to improve adherence and satisfaction with DF CLs.

## METHODS

Twenty‐eight healthy myopic volunteers aged between 18 and 32 years old were enrolled in this comparative, randomised, double‐masked, crossover study. Written consent was obtained after an explanation of the purpose and the protocol of the study. The methodology followed the tenets of the Declaration of Helsinki and was approved by the Ethics Subcommittee for Life and Health Sciences of the University of Minho, Portugal. All subjects had best‐corrected VA of 0.00 logMAR or better in each eye. Subjects with any ocular disease, binocular anomaly, astigmatism >0.75D^7^ or who were taking any medication that contraindicated the use of CLs were excluded from the study. Prior CL wear was not considered. Regular CL wearers were instructed not to wear their CLs for a week before the trials.

### Experimental procedure

The protocol consisted of two visits. At the first visit (baseline visit), case history, subjective refraction with trial lenses, visual acuity and pre‐corneal tear film stability were assessed. The power of the CLs to be used (either −2.00 D, −3.50 D or −5.00 D) was chosen based on the subjective refraction results. Since only three powers were available, the power that was closest to the refraction of the subject was chosen. At the second visit, each pair of DF CLs were fitted (binocularly) in random order and these parameters were assessed in the following order: over‐refraction with trial lenses, quality of vision (QoV) questionnaire, short‐term lens comfort assessment, visual acuity, stereopsis, photopic and mesopic contrast sensitivity, light disturbance assessment, aberrations and pre‐lens tear film stability. Only results from the sensorial dominant eye were included since we aimed to assess the effect of CL design on visual performance without considering other factors such as binocular summation. Nevertheless, both eyes were fitted with CLs to make the conditions as realistic as possible. Sensorial dominance was obtained by means of the ‘+1.50 D blur’ method. Subjects were instructed to look binocularly at the smallest detectable line on the letter chart. A + 1.50D spherical trial lens was held before one eye for a few seconds, removed and then placed before the other eye. The dominant eye was taken as the one experiencing the greater visual disruption.^32^, ^33^

### Dual‐focus contact lenses

**FIGURE 1:**
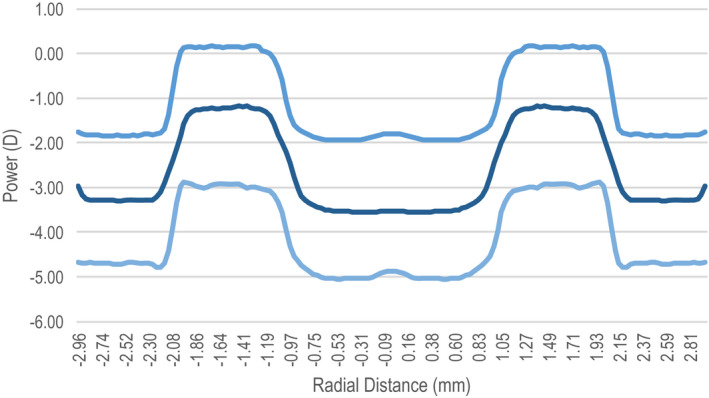
Power profile of the contact lens with the small (2.1 mm) diameter for central distance nominal powers of −2.00 D, −3.50 D and −5.00 D.

**FIGURE 2:**
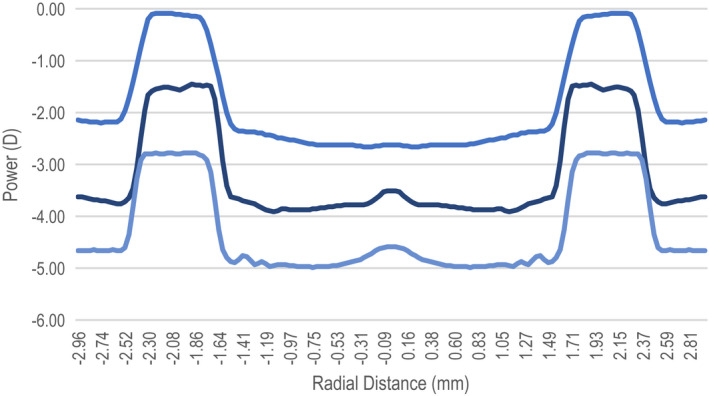
Power profile of the contact lens with the medium (4.0 mm) diameter for central distance nominal powers of −2.00 D, −3.50 D and −5.00 D.

The prototype CLs had a centre distance inner zone of either 2.1 mm or 4.0 mm in diameter, surrounded by alternating zones with +2.0 D add power (Figures 1 and 2).^1^, ^8^, ^14^, ^34^ CL parameters are summarised in Table 1. The lenses were manufactured by Precilens (Precilens, precilens.com). The optical design was the only difference between the CLs. The smaller central diameter (S design) had an inner central zone of 2.1 mm, while the medium central diameter (M design) had an inner zone diameter of 4.0 mm.

**TABLE 1:** Parameters for each contact lens

	Design S	Design M
Inner zone (mm)	2.1	4.0
Add power (D)	+2.0	+2.0
Material	Filcon V3	Filcon V3
Dk	60 units	60 units
Base curve (mm)	8.6	8.6
Lens diameter (mm)	14.0	14.0

*Note:* Dk = permeability; S = contact lens with a small (2.1 mm) central distance diameter; M = contact lens with a medium (4.0 mm) central distance diameter.

The movement and centration of each CL were assessed using a slit lamp. Measurements began 25 min after CL insertion to allow for lens settling.^7^ A wash‐out period of 15 min was allowed between the CLs.^1^, ^35^ During this inter‐lens period, subjects were asked to remain in the laboratory so that differences in light, temperature or humidity did not affect the second CL measurements. At the end of the visit, subjects were asked to choose their preferred CL.

### Measurements

Over‐refraction was performed using an open field binocular autorefractor (Grand Seiko Autorefractometer WAM‐5500, grandseiko.com) and then adjusted subjectively with spherical lenses. The end‐point criterion of maximum plus for best visual acuity was used.^36^ This over‐refraction, in the form of trial lenses, was retained throughout the rest of the protocol, except for the measurement of aberrations. A plano trial lens was introduced if no over‐refraction was needed, so that all subjects were measured under the same conditions. After assessing the over‐refraction, subjects wore these trial lenses for 25 min until the measurements began, since only three CL powers were available.

Subjective quality of vision was assessed using the QoV questionnaire,^32^, ^37^, ^38^ which includes 10 questions about visual symptoms: glare, haloes, starburst, hazy vision, blurred vision, distortion, double or multiple images, fluctuation in vision, focusing difficulties and difficulty in‐depth perception. The questionnaire is scored on a Rasch scale from 0 to 100 depending on the frequency, severity and how bothersome the symptoms were. Lower scores indicate a better subjective quality of vision.^32^, ^37^

General, physical and visual short‐term lens comfort were assessed using continuous visual analogue scales (VAS) between 0 and 10. Lower scores indicate better comfort. Subjects were asked first about the general comfort, followed by physical and visual comfort: ‘How do you rate your overall comfort with the lens?’; ‘How do you rate your physical comfort (pain, foreign body sensation, gritty sensation, dryness, itching…) with the lens?’; ‘How do you rate your visual comfort (blurred vision, distortion or halos, glare and flare around lights) with the lens?’

Photopic visual acuity was measured using the Logarithmic Visual Acuity Chart 2000 (Precision Vision, precision‐vision.com). The Randot Stereotest (Stereo Optical, stereooptical.com) was used to assess stereopsis at 40 cm. Moreover, a Vision Contrast Test System VCTS 6500 was used to measure best‐corrected photopic and mesopic contrast sensitivity at 3 m.^39^ The area under the contrast sensitivity curve was calculated using trapezoidal numerical integration. Photopic conditions were considered ≥3 cd/m^2^ and the mesopic range from 0.01 to 3 cd/m^2^. Pupil diameters were measured from the Grand Seiko autorefractometer WAM‐5500.

Light disturbance is a phenomenon created by the light from a central luminous point causing a halo surrounding the light source. This is an indicator of visual quality. Light disturbance was assessed monocularly using the Light Distortion Analyser (LDA, CEORLab, ceorlab.wixsute.com); a device developed at the Physics Department, University of Minho. It measures the size and shape of the light disturbance surrounding a central bright light source. Several studies have reported that the LDA can assess light disturbance successfully under dim light conditions.^16^, ^17^, ^32^, ^36^, ^40^, ^41^ A detailed description of the system, light sources and measuring procedure can be found in previous work.^32^, ^42^ In the present investigation, semi‐meridians with an angular separation of 30 degrees were measured using an in‐out routine.

Several metrics related to the size and shape of the light disturbance were calculated. The disturbance area was defined as the sum of the semi‐meridian areas assessed in mm^2^. The Light Disturbance Index (LDI) expresses the percentage of the total area covered by the light distortion and is the ratio of the area of points missed by the subject to the total area explored. The best‐fit circle radius describes the circle that best fits the disturbance area, expressed in millimetres. The deviation of the obtained polygonal shape from the best‐fit circle is the best‐fit circle irregularity, while the standard deviation of the best‐fit circle irregularity measures the asymmetry of the light disturbance shape from the perfect circular shape of the best‐fit circle and indicates the light disturbance irregularity.^32^, ^36^ Pupil diameter was measured in the contralateral eye while subjects were performing the task using a NeurOptics® VIP™‐200 Pupillometer (NeurOptics, neuroptics.com).

A Hartmann‐Shack Aberrometer (irx3™, Imagine Eyes, imagine‐eyes.com) was used to measure ocular aberrations and reconstruct them using Zernike polynomials for pupil diameters of 3 and 5 mm. These diameters were chosen in line with previous studies.^43^, ^44^, ^45^ Root mean square (RMS) was calculated with the CL in situ for lower‐order aberrations (LOAs), higher‐order aberrations (HOAs) up to the 9th order and total aberrations.

Finally, the Medmont E 300, version 6.1 (Medmont, medmont.com.au) was used to assess Tear Film Surface Quality (TFSQ). TFSQ is a previously validated algorithm, which has been reported to predict tear film stability^46^ and to be able to discern between dry and non‐dry eyes.^47^ It analyses the structure of the Placido disk pattern reflected onto the tear film after blinking over time. TFSQ values range from 0 to 1. A value ≥0.30 indicates a destabilised tear film with distortions in the ring pattern. Greater TFSQ scores represent a less regular tear film.^46^, ^47^ Three metrics are automatically calculated by the device: TFSQ, TFSQ area and auto Tear Break‐Up Time. TFSQ area corresponds to the percentage of the area assessed with a TFSQ value >0.30, while auto Tear Break‐Up Time is the time in seconds in which the TFSQ area is at least 5.0% in two consecutive images.^46^, ^47^ Tear film stability was measured for 30 s on three consecutive occasions, and the mean and median were calculated for each measurement. Room temperature and humidity remained stable during all visits.

Luminance and illuminance were evaluated using a luminance meter (LS‐100, Konica Minolta, konicaminolta.com) and an illuminance meter (T‐10, Konica Minolta, konicaminolta.com), respectively. The varying daylight was removed by using blackout curtains in the examination room. Room illuminance was 255.58 ± 8.22 and 3.52 ± 0.12 lux under photopic and mesopic conditions, respectively. The mean luminance was 203.10 ± 3.68 cd/m^2^, 48.09 ± 1.15 cd/m^2^ and 0.63 ± 0.01 cd/m^2^ for the measurement of visual acuity, photopic and mesopic contrast sensitivity at 3 m, respectively.

### Statistical analysis

SPSS v26.0 statistical software for Microsoft Windows (IBM, ibm.com) was used to perform the statistical analysis. The Shapiro–Wilk test was used to check data for normality. Results were reported as median and interquartile ranges if they were not normally distributed.

Differences between the two CLs for each parameter were evaluated with the paired t‐test or Wilcoxon signed‐rank test, depending upon the sample distribution. Differences between each CL and baseline for the tear film analysis were evaluated using either analysis of variance (ANOVA) or the Friedman test. *Post‐hoc* pairwise comparisons were carried out using Bonferroni correction. The interaction between CL type and the order in which they were fitted was assessed by means of a mixed ANOVA. *P*‐values < 0.05 were considered statistically significant.

## RESULTS

Twenty‐eight eyes from 28 subjects (17 female and 11 male) were included, 20 of whom were CL wearers. The mean age was 23.5 ± 4.1 years, ranging from 18 to 32 years. Median spherical refraction was −1.00 D (interquartile range: −2.31 to −0.27 D), while median astigmatism was 0.00 D (interquartile range: −0.50 to 0.00 D).

Eighteen subjects were fitted with a CL having a distance nominal power of −2.00 D, six with −3.50 D and four with −5.00 D. After lens fitting, the median spherical over‐refraction was +1.00 D (interquartile range: +0.50 to +1.25 D) and +1.00 D (interquartile range: +0.56 to +1.25 D) for the small and medium diameter inner optic zones, respectively. The CLs were considered to have acceptable movement, centration and coverage in all subjects.

### Visual acuity, stereopsis and contrast sensitivity

Distance best‐corrected visual acuity, stereopsis and best‐corrected contrast sensitivity under photopic and mesopic conditions for each CL design are shown in Table 2. The majority of subjects (60.7%) wearing the S design achieved stereopsis of 20 s of arc and 39.3% achieved between 20 and 25 s of arc. With the M design, 50% of subjects achieved stereopsis of 20 s of arc, 46.4% between 20 and 25 s of arc and 3.6% between 25 and 40 s of arc.

**TABLE 2:** Best‐corrected distance visual acuity, stereopsis and best‐corrected photopic and mesopic contrast sensitivity for each CL design

Measurement	Condition	Median (Interquartile range)	Significance level (Statistic, *p*‐value)
Best‐corrected distance visual acuity (LogMAR)	S	−0.05 (0.00 to −0.10)	(t_27_ = −0.98, *p* = 0.38)^a^
	M	−0.10 (−0.02 to −0.10)	
Stereopsis (seconds of arc)	S	22.0 (20 to 25)	(Z_27_ = 1.51, *p* = 0.13)^b^
	M	22.5 (20 to 25)	
Photopic contrast sensitivity 1.5 cpd (db)	S	35 (35 to 70)	(Z_27_ = −1.14, *p* = 0.25)^b^
	M	35 (23.75 to 35)	
Photopic contrast sensitivity 3 cpd (db)	S	85 (85 to 170)	(Z_27_ = −1.04, *p* = 0.30)^b^
	M	85 (54.25 to 170)	
Photopic contrast sensitivity 6 cpd (db)	S	45 (45 to 111.25)	(Z_27_ = 1.14, *p* = 0.26)^b^
	M	70 (45 to 70)	
Photopic contrast sensitivity 12 cpd (db)	S	23.50 (15 to 32)	(Z_27_ = 0.79, *p* = 0.43)^b^
	M	32 (15 to 55)	
Photopic contrast sensitivity 18 cpd (db)	S	5.50 (4 to 10)	(Z_27_ = 3.88, *p* < 0.001)^b^ ^,^ ^c^
	M	10 (10 to 15)	
Area under the photopic contrast sensitivity curve	S	577.5 (522 to 1157.63)	(Z_27_ = 1.02, *p* = 0.35)^b^
	M	754.5 (462.38 to 1098.75)	
Mesopic contrast sensitivity 1.5 cpd (db)	S	20 (20 to 20)	(Z_27_ = 0.12, *p* = 0.90)^b^
	M	20 (20 to 20)	
Mesopic contrast sensitivity 3 cpd (db)	S	24 (24 to 44)	(Z_27_ = −0.97, *p* = 0.33)^b^
	M	24 (24 to 44)	
Mesopic contrast sensitivity 6 cpd (db)	S	11 (6.5 to 21)	(Z_27_ = −1.63, *p* = 0.10)^b^
	M	21 (11 to 21)	
Mesopic contrast sensitivity 12 cpd (db)	S	5 (0 to 5)	(Z_27_ = 0.29, *p* = 0.77)^b^
	M	5 (5 to 7.25)	
Mesopic contrast sensitivity 18 cpd (db)	S	0 (0 to 4)	(Z_27_ = 1.64, *p* = 0.10)^b^
	M	0 (0 to 4)	
Area under the photopic contrast sensitivity curve	S	148.5 (98.25 to 250.5)	(Z_27_ = 1.08, *p* = 0.38)^b^
	M	193.5 (148.5 to 264.0)	

*Note:* S = contact lens with the small (2.1 mm) central distance ring diameter; M = contact lens with the medium (4.0 mm) central distance ring diameter.

^a^ *T*‐test.

^b^ Wilcoxon.

^c^ Statistically significant differences; cpd = cycles per degree; dB = decibel.

No significant differences were found between the two DF CLs for visual acuity, stereopsis, photopic and mesopic contrast sensitivity, except for photopic contrast sensitivity at a spatial frequency of 18 cycles per degree (cpd) (*p* < 0.001), where contrast sensitivity was higher with the M design.

**FIGURE 3:**
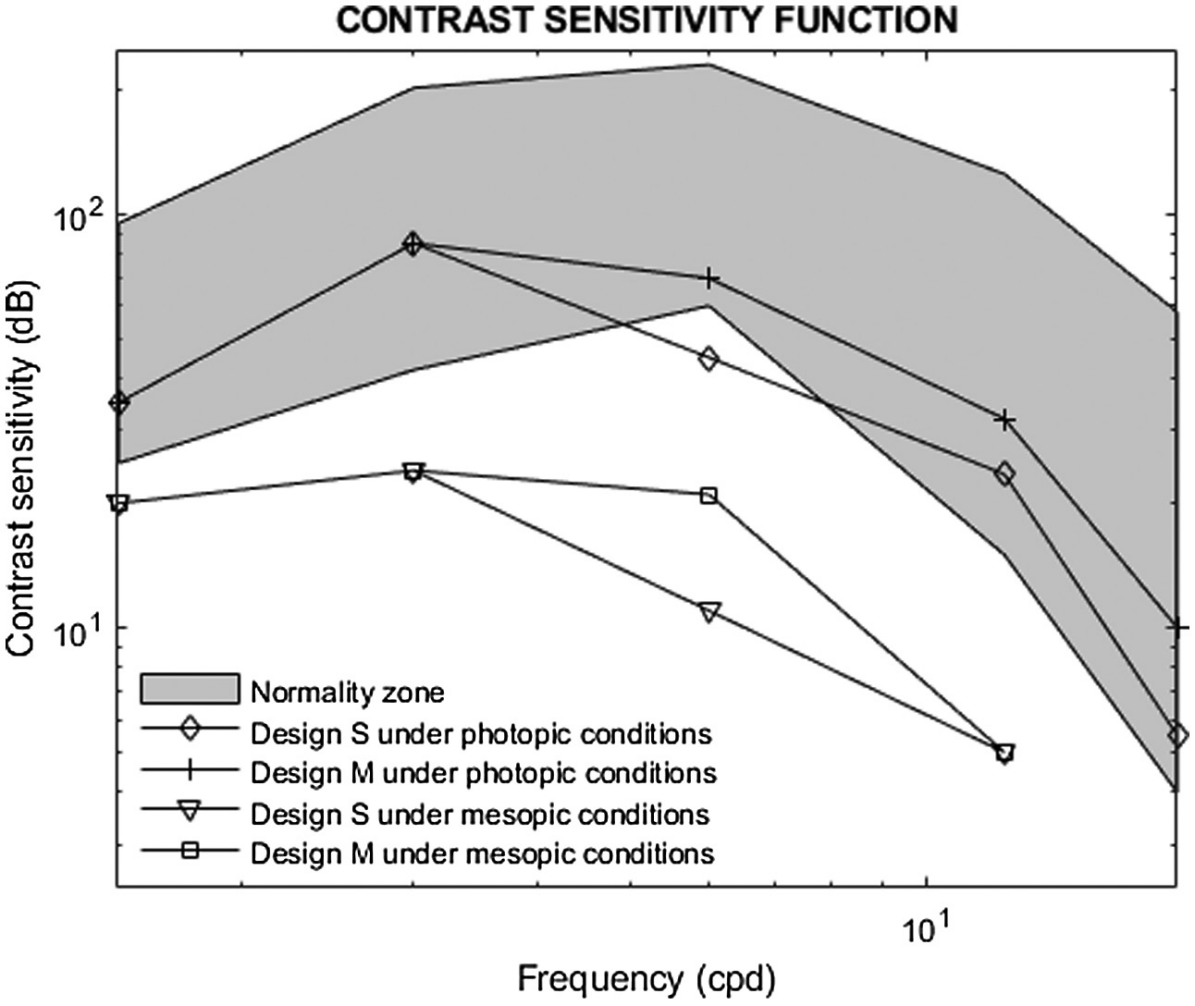
Best‐corrected contrast sensitivity function for each contact lens design under photopic and mesopic conditions. S = small (2.1 mm) central diameter; M = medium (4.0 mm) central diameter.

The contrast sensitivity function followed a normal physiological shape with the highest sensitivity at 3 cpd (Figure 3). Both CLs showed similar performance under both photopic and mesopic conditions. Photopic contrast sensitivity curves were within the normality zone^39^ for all spatial frequencies, except for the S design at 6 cpd. However, mesopic contrast sensitivity fell below the normality zone at all spatial frequencies with both CLs.

The mean pupil diameter under photopic conditions was 4.44 ± 0.43 mm and 4.45 ± 0.42 mm for the S and M designs, respectively (t_27_ = −0.96, *p* = 0.39). The mean pupil diameter under mesopic conditions was 6.39 ± 0.38 mm and 6.36 ± 0.35 for the S and M designs, respectively (t_27_ = −1.16, *p* = 0.24). Pupil diameter was significantly larger under mesopic conditions for the S (t_2 7_ = −13.73, *p* < 0.001) and M design (t_27_ = −16.92, *p* < 0.001).

### Light disturbance

Table 3 shows light distortion findings for each CL design. Disturbance area, light distortion index and best‐fit circle radius were significantly higher for the S design than for the M design (*p* ≤ 0.02). Nevertheless, no significant differences between the CLs were found in the irregularity of the light distortion (best‐fit circle irregularity and standard deviation of best‐fit circle irregularity). Mean pupil diameter for the S and M CLs while subjects were being assessed for LDA was 5.20 ± 0.76 and 5.15 ± 0.79 mm, respectively (t_27_ = 0.29, *p* = 0.82).

**TABLE 3:** Light disturbance for each CL

Measurement	Lens	Median (Interquartile range)	Significance level (Statistic, *p*‐value)
Disturbance area (mm^2^)	S	3384 (2396–5504)	(Z_27_ = −2.46, *p* = 0.01)^a^ ^,^ ^b^
	M	2744 (1852–4148)	
Light distortion index (%)	S	16.83 (11.92–27.38)	(Z_27_ = −2.44, *p* = 0.01)^a^ ^,^ ^b^
	M	13.65 (9.21–20.63)	
Best‐fit circle radius (mm)	S	33.30 (28.18–42.65)	(Z_27_ = −2.39, *p* = 0.02)^a^ ^,^ ^b^
	M	30.00 (24.85–36.98)	
Best‐fit circle irregularity (mm)	S	0.71 (0.37–0.98)	(Z_27_ = −0.08, *p* = 0.94)^b^
	M	0.79 (0.34–1.03)	
Best‐fit circle irregularity SD (mm)	S	4.86 (4.06–7.65)	(Z_27_ = −1.48, *p* = 0.14)^b^
	M	4.78 (3.15–5.95)	

*Note:* S = contact lens with the small (2.1 mm) central distance ring diameter; M = contact lens with the medium (4.0 mm) central distance ring diameter.

^a^ Wilcoxon.

^b^ Statistically significant differences; mm = millimetres.

### Wavefront aberrations

Table 4 shows LOA, HOA and total aberrations with 3 and 5 mm pupil diameters for each CL. No significant differences between CLs were found for LOA and total aberrations. For the CL measurements, subjects were corrected with a lens of similar power to the subject's refractive error, i.e., −2.00 D, −3.50 D or −5.00 D. Thus, comparisons of both LOA and total aberrations were related to the residual refractive error of the subjects.^48^ However, HOA were not related to the residual refractive error and therefore can be used to compare the aberrations induced by the DF design. The M design showed statistically higher levels of HOA for a 5 mm pupil diameter, compared with the S design. No other significant differences were observed between the lenses.

**TABLE 4:** Lower‐order, higher‐order and total aberrations for pupil diameters of 3 and 5 mm for each condition

Measurement	Condition	Median (Interquartile range)	Significance level (Statistic, *p*‐value)
Lower‐order 3 mm RMS (μm)	S	0.30 (0.19–0.39)	(Z_27_ = 1.79, *p* = 0.08)^a^
	M	0.37 (0.27–0.50)	
Higher‐order 3 mm RMS (μm)	S	0.23 (0.19–0.25)	(t_27_ = 0.47, *p* = 0.64)^b^
	M	0.22 (0.17–0.27)	
Total aberrations 3 mm RMS(μm)	S	0.38 (0.31–0.45)	(Z_27_ = 1.82, *p* = 0.07)^a^
	M	0.44 (0.32–0.52)	
Lower‐order 5 mm RMS (μm)	S	0.55 (0.36–0.81)	(Z_27_ = 0.44, *p* = 0.66)^a^
	M	0.42 (0.30–0.93)	
Higher‐order 5 mm RMS (μm)	S	0.37 (0.33–0.42)	(Z_27_ = 3.81, *p* < 0.001)^a^ ^,^ ^b^
	M	0.51 (0.44–0.54)	
Total aberrations 5 mm RMS (μm)	S	0.63 (0.55–0.89)	(Z_27_ = 1.92, *p* = 0.06)^a^
	M	0.69 (0.60–1.05)	

*Note:* S = contact lens with the smaller (2.1 mm) central distance ring diameter; M = contact lens with the medium (4.0 mm) central distance ring diameter.

^a^ Wilcoxon.

^b^ *T*‐test.

^c^ Statistically significant differences; μm = micrometres; RMS = root mean square.

### Tear film stability

Results for tear film stability at baseline (pre‐corneal tear film stability) and for each CL (pre‐lens tear film stability) are shown in Table 5. Tear film stability was poorer when subjects were wearing either CL (higher TFSQ and TFSQ area and lower auto Tear Break‐Up Time) when compared with baseline. However, no significant differences were found between the CLs for the tear film stability, which suggests that variations in the diameter of the CL inner zone do not cause changes in tear film stability.

**TABLE 5:** Tear Film Surface Quality (TFSQ) metrics for each experimental condition

Measurement	Condition	Median (Interquartile range)	Significance level (Statistic, *p*‐value)	Post‐hoc (*p*‐value)
Mean TFSQ	Baseline	0.13 (0.08–0.20)	<0.001^a^ ^,^ ^c^	Baseline‐S: <0.001^b^ ^,^ ^c^
	S	0.37 (0.26–0.45)		Baseline‐M: <0.001^b^ ^,^ ^c^
	M	0.38 (0.31–0.51)		S‐M: 0.54^b^
Median TFSQ	Baseline	0.10 (0.07–0.17)	<0.001^a^ ^,^ ^c^	Baseline‐S: <0.001^b^ ^,^ ^c^
	S	0.36 (0.23–0.43)		Baseline‐M: <0.001^b^ ^,^ ^c^
	M	0.38 (0.32–0.53)		S‐M: 0.54^b^
Mean TFSQ area (%)	Baseline	7.17 (1.44–14.37)	<0.001^a^ ^,^ ^c^	Baseline‐S: <0.001^b^ ^,^ ^c^
	S	32.20 (23.84–46.18)		Baseline‐M: <0.001^b^ ^,^ ^c^
	M	37.10 (27.20–52.36)		S‐M: 1.00^b^
Median TFSQ area (%)	Baseline	4.10 (0.87–12.85)	<0.001^a^ ^,^ ^c^	Baseline‐S: <0.001^b^ ^,^ ^c^
	S	32.90 (21.78–44.75)		Baseline‐M: <0.001^b^ ^,^ ^c^
	M	38.45 (25.60–54.08)		S‐M: 0.43^b^
Mean auto tear break‐up time (seconds)	Baseline	7.12 (4.73–11.46)	<0.001^a^ ^,^ ^c^	Baseline‐S: <0.001^b^ ^,^ ^c^
	S	2.54 (2.40–2.60)		Baseline‐M: <0.001^b^ ^,^ ^c^
	M	2.47 (2.40–2.56)		S‐M: 1.00^b^
Median auto tear break‐up time (seconds)	Baseline	6.89 (4.73–10.74)	<0.001^a^ ^,^ ^c^	Baseline‐S: <0.001^b^ ^,^ ^c^
	S	2.50 (2.40–2.60)		Baseline‐M: <0.001^b^ ^,^ ^c^
	M	2.50 (2.40–2.60)		S‐M: 1.00^b^

*Note:* Baseline = no lens present; S = contact lens with an inner zone diameter of 2.1 mm; M = contact lens with an inner zone diameter of 4.0 mm;

^a^ Friedman.

^b^ Bonferroni.

^c^ Statistically significant differences; TFSQ = tear film surface quality.

### Questionnaires

Table 6 shows the results from the QoV questionnaire. No significant differences were found between the two CLs for QoV values in the frequency, severity and bothersome subscales. Table 7 shows the results for short‐term lens comfort. Scores did not differ significantly between the CL designs for overall, physical and visual short‐term lens comfort. Finally, 13 subjects (46.4%) preferred the S design CL, whilst 15 subjects (53.6%) preferred the M design CL.

**TABLE 6:** Quality of Vision questionnaire scores for each contact lens

Measurement	Lens	Median (Interquartile range)	Significance level (Statistic, *p*‐value)
QoV frequency score	S	49 (42–59)	(Z_27_ = −0.99, *p* = 0.32)^a^
	M	49 (37–52)	
QoV severity score	S	42 (35–48.5)	(Z_27_ = −1.16, *p* = 0.25)^b^
	M	39 (32–47)	
QoV bothersome score	S	42 (29–53)	(t_27_ = 0.38, *p* = 0.70)^a^
	M	38 (29–49)	

*Note:* S = contact lens with an inner zone diameter of 2.1 mm; M = contact lens with an inner zone diameter of 4.0 mm; QoV = Quality of Vision.

^a^ Wilcoxon.

^b^ *T*‐test.

**TABLE 7:** Visual analogue scale for short‐term lens comfort for each contact lens

Measurement	Lens	Median (Interquartile range)	*p*‐Value
Overall short‐term lens comfort score	S	3.00 (2.00–4.00)	0.33^a^
	M	3.50 (2.00–5.00)	
Physical short‐term lens comfort score	S	3.00 (2.00–4.00)	0.89^a^
	M	3.00 (2.00–4.00)	
Visual short‐term lens comfort score	S	4.00 (3.00–5.75)	0.26^a^
	M	4.00 (3.00–4.75)	

*Note:* S = contact lens with an inner zone diameter of 2.1 mm; M = contact lens with an inner zone diameter of 4.0 mm.

^a^ Wilcoxon.

No significant interaction was found between CL type and the order in which the lenses were fitted for any of the parameters reported here (all *p* > 0.42). Thus, the order of lens fitting was not a confounding factor in the results.

## DISCUSSION

DF CLs are pupil‐dependent, and their performance is influenced by the ratio between the distance and added power zones, and the area of the pupil exposed to these zones.^8^, ^19^, ^20^, ^21^, ^22^, ^23^, ^24^, ^25^, ^49^, ^50^ Thus, wider central diameters have the added power located further away from the centre of the visual field, which causes less impairment to central vision.^2^ To investigate the influence of the inner zone diameter on visual performance, two prototype DF CLs designed to reduce myopia progression were assessed in this study.

### Visual acuity, stereopsis and contrast sensitivity

The results demonstrate that both CLs provided excellent visual acuity and stereopsis, with no significant differences between the lenses. Values of visual acuity, stereopsis and contrast sensitivity were similar to the results found in a previous study with a different DF CL.^16^ Talens‐Estarelles et al.^20^ and Martins et al.^1^ reported better distance visual acuity in CLs with larger areas for distance vision. Further, Przekoracka et al.^2^ found that a myopia control CL with a central zone of 3 mm, +4.00 D Add and a polynomial progression zone reduced distance visual acuity in comparison with a CL with a 4.5 mm central zone. However, they did not observe any reduction with a +2.00 Add. Additionally, the authors found that both diameters affected contrast sensitivity by the same amount, which suggests that even medium‐distance central diameters may impair contrast sensitivity. These results are in line with the results of the present study using a +2.00 D Add. Therefore, visual acuity might have been affected had a higher add power been used. However, these studies are not directly comparable due to differences in methodology and lenses used.

Photopic contrast sensitivity curves lay inside the normality zone^39^ for all spatial frequencies except for the S design at 6 cpd. Nevertheless, young subjects were expected to lie near the top of the normality zone. Photopic contrast sensitivity for the 18 cpd spatial frequency was better with the M design (*p* < 0.001). This is in accordance with the results of Martins et al.^1^ and Talens‐Estarelles et al.,^20^ who found that subjects fitted with designs having larger inner zone diameters showed better contrast sensitivity at both medium and high spatial frequencies.

Various studies have found poorer contrast sensitivity at high spatial frequencies in eyes fitted with multifocal CLs.^1^, ^2^, ^16^, ^35^, ^51^, ^52^ Considering that both DF and multifocal CLs have zones of power that overcorrect the distance refractive error, these superimposed out‐of‐focus images will create veiling on the retina and decrease contrast modulation.^21^, ^52^, ^53^ Therefore, it is expected to find a better quality of vision at distance with designs that have a higher contribution to the distance correction.

### Light disturbance

Previous studies^1^, ^32^, ^41^ found more light disturbance with multifocal compared with single‐vision CLs, which tended to worsen with lenses having smaller zones for distance vision. In this study, the size of the light disturbance was greater for the S design. Nevertheless, the shape of the light disturbance was not affected. These findings are aligned with a previous study^1^ that found less light disturbance with myopia control lenses having larger areas for distance vision, without differences in the shape of the halo. This might be explained by the fact that lenses with larger areas for distance vision send a greater percentage of light to the distance focus, thereby reducing the amount of out‐of‐focus light, which translates into less light disturbance and increased contrast modulation. However, the shape of the light disturbance could remain unaltered because both lenses have circular rings of the same shape.

Although not directly comparable, the CL with the medium inner zone diameter showed similar light disturbance to a myopia control DF CL assessed in a previous study.^16^ Nevertheless, the design with the smaller inner zone diameter showed greater light disturbance compared with the results found in the previous investigation.

### Wavefront aberrations

Previous studies^1^, ^5^, ^16^, ^54^, ^55^ found that DF and multifocal CLs induce higher levels of HOA due to the concentric zones of increasing power. The medium inner zone diameter lens showed higher levels of HOA for a 5 mm pupil diameter. This does not correlate with our light disturbance findings, where subjects fitted with the M design reported less light disturbance compared with the S design.

This might be explained as the M lens has a more progressive power design than the S lens. In opposition to these findings, Martins et al.^1^ found lower HOA in lenses with larger areas for distance vision. Moreover, it has also been reported that the image quality of CLs with large central diameters is less affected by spherical aberration.^8^ HOA in the present study were similar to those found in a previous investigation using a different myopia control CL.^16^ It is worth noting that due to the abrupt changes in the power profiles of both designs, these wavefronts might not be best represented by a Zernike expansion.^48^

### Tear film stability

Tear film stability did not show significant differences between the CLs, suggesting that changes in the diameter of the zones did not affect pre‐lens tear film spreading and stability across the CL surface. To our knowledge, only one previous study measured pre‐lens tear film stability with a DF CL.^28^ They found decreased tear film stability with a DF CL compared with a single‐vision CL made from the same material. This might be caused by the decreased spreading of the pre‐lens tear film across the concentric ring pattern. In comparison with this previous work, tear film stability was similar to or slightly better with the CLs assessed in the current investigation.

The present study assessed tear film stability 25 minutes after lens insertion. However, tear film stability and comfort might change following longer periods of CL wear. Nevertheless, other investigations reported that this period is adequate to evaluate the short‐term performance of CLs.^56^, ^57^, ^58^, ^59^ The tear film changes during the first 20 min following lens insertion. Efron et al.^57^ reported that the majority of lens dehydration occurred in the first 5 min. However, other authors claim that tear film changes over the first 30 min.^60^, ^61^ Therefore, there is still controversy regarding this topic. Nevertheless, this should not alter the comparison between the two CLs, since each was assessed after the same time and wash‐out period. Furthermore, randomising the order of lens fitting helps avoid bias.

### Questionnaires

No significant differences were found between the two CLs for the QoV scores. This suggests that despite the CL with the smaller central diameter inducing more light disturbance and lower contrast sensitivity at higher spatial frequencies, short‐term, subjective visual lens comfort was not affected. This fact might be explained since light disturbance was measured under dim light conditions, which differs from normal lighting. In comparison with two previous studies^16^, ^28^ using a different, myopia control DF CL (MiSight), QoV scores, overall and physical comfort scores were similar, although short‐term lens comfort was worse in the present investigation. Additionally, binocular summation and the process of neuroadaptation might improve these values in a longer follow‐up trial.^17^, ^32^, ^62^, ^63^ These results might also be applied to subjects with presbyopia, due to the similarities between DF CLs and multifocal CLs.

Apart from those mentioned above, the present study has other limitations to be considered. The Vision Contrast Test System VCTS 6500, used to assess contrast sensitivity, has been reported to have poor repeatability.^64^ In addition, the Randot Stereotest only measures stereopsis down to 20 seconds of arc, which is the expected value for 50% of a visually‐normal population.^65^ Moreover, tests used in this study challenged the visual system since they were performed in dim light or glare conditions, which are not typical conditions for children. Therefore, the results might underestimate the performance of the DF CLs under normal conditions in children. A prior history of CL wear was not taken into account, and some of the subjects were new CL wearers. The short‐term visual and physical comfort of the CLs could have been underestimated, since neophyte CL wearers are less accustomed to wearing lenses. Further, only the quality of vision at distance was assessed. Future research should also include the analysis of visual performance at near.

Furthermore, as the CLs were prototypes, only three different dioptric powers were available. Trial lenses were placed in front of the participants' eyes to correct the over‐refraction, except for the measurement of aberrations. This might impact both visual and optical quality. However, the aim was not to assess the real visual performance of these lenses, but rather to compare the two designs and to assess whether the central diameter of a DF CL has an effect on visual performance and tear film stability. Therefore, the comparison of the two lenses should not be influenced by the power of the contact lens or the over‐refraction. Indeed, previous studies have also used a similar methodology with trial lenses to assess the visual and optical quality of other CLs.^16^, ^28^ Further studies are needed to assess each CL having the correct power. Nevertheless, the present work still allows for building a hypothesis.

In addition, baseline measurements were performed with the naked eye. It would have been better to use a single‐vision contact lens for a direct comparison with the DF CLs. Finally, the study was performed on a young adult sample with high visual and comfort demands, who might not be representative of children.^66^

Overall, the present study may help clinicians to adjust the lens design depending on patients' requirements to improve their adherence and satisfaction with DF CLs. Both CLs tested here provided acceptable visual performance under photopic conditions. The lens with the medium central inner zone provided better photopic contrast sensitivity with high spatial frequencies and induced less light disturbance. However, the lens with the smaller central zone diameter induced less HOA for a pupil diameter of 5 mm. Despite the variations in visual quality, no differences in short‐term visual lens comfort were found between the lens designs, which suggests that each diameter gave rise to the same subjective quality of vision. No differences were found between the two designs for visual acuity, stereopsis, mesopic contrast sensitivity, light disturbance shape and HOA with a 3 mm pupil diameter. Further studies are needed to assess the effect of these CLs on visual performance with the correct refractive power, a longer follow‐up time and to compare the results with a single‐vision CL correction. Other paths to deepen knowledge in this field will eventually require the use of other lens designs, different materials, different pupil size conditions and investigation of the effects under different lighting and viewing distances.

## AUTHOR CONTRIBUTIONS

**José Vicente García‐Marqués:** Conceptualization (equal); data curation (equal); formal analysis (equal); funding acquisition (equal); investigation (equal); methodology (equal); resources (equal); writing – original draft (equal). **Rute J Macedo‐de‐Araújo:** Conceptualization (equal); data curation (equal); formal analysis (equal); funding acquisition (equal); investigation (equal); resources (equal); supervision (equal); writing – review and editing (equal). **Colm McAlinden:** Formal analysis (equal); investigation (equal); methodology (equal); supervision (equal); validation (equal); writing – review and editing (equal). **Miguel Faria‐Ribeiro:** Conceptualization (equal); data curation (equal); formal analysis (equal); investigation (equal); methodology (equal); resources (equal); software (equal); supervision (equal); validation (equal); writing – review and editing (equal). **Alejandro Cerviño:** Conceptualization (equal); funding acquisition (equal); investigation (equal); methodology (equal); project administration (equal); supervision (equal); writing – review and editing (equal). **José Manuel González‐Méijome:** Conceptualization (equal); data curation (equal); funding acquisition (equal); investigation (equal); methodology (equal); project administration (equal); resources (equal); software (equal); supervision (equal); validation (equal); writing – review and editing (equal).

## CONFLICT OF INTEREST

José Manuel González Méijome and Rute J Macedo‐de‐Araújo have a proprietary interest in the light disturbance analyser.
